# Designing a Substance Misuse Data Dashboard for Overdose Fatality Review Teams: User-Centered Design Approach

**DOI:** 10.2196/79407

**Published:** 2026-01-15

**Authors:** Marie Pisani, Madeline K Oguss, Julia Dickson-Gomez, Constance Kostelac, Amy Parry, Starr Moss, Elizabeth Salisbury-Afshar, Brian Patterson, Michael Spigner, Megan Gussick, Alison Krautkramer, Timothy Gruenloh, Askar Safipour Afshar, Preeti Gupta, Anoop Mayampurath, Majid Afshar

**Affiliations:** 1 Department of Medicine, University of Wisconsin, Madison, WI, United States; 2 School of Medicine and Public Health, University of Wisconsin, 610 Walnut St, Suite 517, Madison, WI, 53726, United States, 1 6082636400; 3 Institute for Health and Equity, Medical College of Wisconsin, Milwaukee, WI, United States; 4 United States Department of Justice, Madison, WI, United States; 5 Department of Family Medicine and Community Health, University of Wisconsin, Madison, WI, United States; 6 BerbeeWalsh Department of Emergency Medicine, University of Wisconsin, Madison, WI, United States; 7 Department of Biostatistics and Medical Informatics, University of Wisconsin, Madison, WI, United States; 8 Division of Pulmonary, Critical Care, Sleep, and Allergy, University of Illinois Chicago, Chicago, IL, United States

**Keywords:** data linkage, overdose, public health informatics, qualitative research, substance use disorders

## Abstract

**Background:**

Overdose fatality review (OFR) is a public health process in which cases of fatal overdose are carefully reviewed to identify prevention strategies. Current OFR requires review of multiple unconnected data sources, which is a manually intensive process. The Substance Misuse Data Commons (SMDC) was created to link electronic health record data with data from local and state agencies into a single, cloud-based e-platform but does not currently have a data visualization tool.

**Objective:**

We aimed to use human factors design principles to develop a comprehensive dashboard for the SMDC that could facilitate enhanced processes to support OFR.

**Methods:**

We first surveyed OFR leaders in Wisconsin using the National Aeronautics and Space Administration-Task Load Index to understand the cognitive workload of 3 tasks: (1) analysis of population-level overdose trends, (2) selection and preparation of individual cases for review, and (3) abstraction of data from individual causes. We then conducted semistructured interviews to identify targets for workflow optimization. Next, we developed a prototype dashboard for evaluation using a synthetic dataset built with GPT-4. We subsequently performed iterative design sessions with heuristic evaluations and collected end-user feedback on the final prototype via a second round of semistructured interviews and targeted surveys, including the Unified Theory of Acceptance and Use of Technology and the Perceived Usefulness Questionnaire.

**Results:**

The National Aeronautics and Space Administration-Task Load Index revealed a moderately high mental workload with the current workflow for all 3 tasks, with mean scores of 12.60 (SD 3.31), 11.90 (SD 3.57), and 12.43 (SD 5.41) for tasks 1, 2, and 3, respectively. Interviews pointed to causes including technological challenges and a reliance on manual processes. The prototype dashboard addressed these concerns by integrating multiple data sources to generate population-level visualizations and patient-level event timelines. End users reported the potential for improved efficiency and data accessibility compared to antecedent processes. The Unified Theory of Acceptance and Use of Technology results indicated the dashboard would likely be adopted if made available, with a mean of 4.07 out of 5.00 (SD 0.65). The Perceived Usefulness Questionnaire results suggested moderate usefulness for both the aggregate and individual-level data, with means of 3.61 (SD 0.82) and 3.64 (SD 0.85) out of 5.00, respectively.

**Conclusions:**

OFR is a data-intensive process that traditionally demands substantial cognitive and manual effort, and there are multiple barriers to efficiently collecting data and presenting them for review. The dashboard offers a user-centered, informatics-based approach to streamline data aggregation and presentation, potentially enhancing the efficiency of case reviews. Implementing a dashboard that consolidates and visualizes disparate data sources has the potential to alleviate the manual workload in OFR. Ultimately, our aim is to deliver a finalized data dashboard with real-world SMDC data, giving OFR leaders additional tools to aid in their rigorous work shaping interventions to reduce overdose fatalities.

## Introduction

Drug overdose death rates have been steadily rising over the past 2 decades, with the most significant annual spike occurring between 2019 and 2020, when rates surged by 31.0% in the United States [1]. To address this public health crisis, some local health departments around the country have assembled overdose fatality review (OFR) teams. OFR teams are multidisciplinary and multiagency teams with representatives from areas such as public health, safety, social services, medical examiners and coroners, emergency responders, substance use treatment providers, and other community stakeholders [2]. These teams discuss the local trends in overdose fatalities and review individual cases to identify and implement recommendations aimed at preventing overdose deaths [2]. OFR teams can help facilitate harm reduction strategies such as syringe services programs, naloxone education and training, outreach programs, and coordination of treatment services from health care settings [3]. However, many counties have yet to implement OFRs, and those that exist exhibit considerable heterogeneity in their data collection procedures, requiring substantial effort in data curation.

OFR teams in Wisconsin perform fatality reviews by integrating population- and individual-level data. These reviews involve an in-depth exploration of an individual’s timeline before their death, focusing on potential opportunities for intervention. The data may come from different sources, including the medical examiners and coroners office, emergency medical services (EMS), the Department of Corrections, law enforcement, social media, and other local and state agencies. Often, obtaining these data requires collecting data on individual cases from each agency and linking across multiple sources.

The Substance Misuse Data Commons (SMDC) is a single, cloud-based data repository that links hospital electronic health record (EHR) data for patients with substance misuse to local and state agency data from EMS, the Department of Corrections, the Prescription Drug Monitoring Program, state and national death sources, statewide medical and pharmacy claims, and neighborhood-level socioeconomic data [4]. It was recently created by our team to address the issue of siloed datasets for substance misuse research, but it currently lacks an interface for data visualization [4]. Dashboards have been recognized as effective tools for visualizing public health data and facilitating disease surveillance, targeted analyses, and decision-making [5]. Additionally, a well-designed dashboard can reduce cognitive workload and improve efficiency by decreasing the amount of time spent gathering data [6]. In several states, such as Indiana and North Carolina, health departments have used dashboards of aggregate data to monitor overdose trends and uncover preventable risk factors [7,8]. While these dashboards are effective for tracking local and state-wide trends and setting case review priorities, they often lack the breadth of data and the level of detail needed by OFR teams. Currently, OFR teams in Wisconsin do not have access to the SMDC. Their data collection processes rely on manually requesting and compiling information from multiple partner agencies. The SMDC was established as a research infrastructure to enable secure multiagency data linkage, and this study represents the first effort to design a visualization interface that could make those linked data accessible and actionable for OFR teams in the future.

The objective of Phase 1 of this study was to assess the cognitive workload of OFR teams and their currently utilized data processes. The objective of Phase 2 of this study was to design a prototype data dashboard and then assess the usability and acceptability of the dashboard in a simulated study. This study introduces a novel, user-centered approach to understanding and improving the OFR process by combining cognitive workload assessment with prototype dashboard design. Unlike prior public health surveillance dashboards, our approach integrates multiagency data within a unified framework modeled on real OFR workflows and leverages synthetic data generation using large language models to enable privacy-preserving development and testing.

## Methods

### Phase 1: Analysis of Current Workflow

We conducted surveys and semistructured interviews with OFR leaders in Wisconsin to document the current workflow and associated cognitive workload for data collection and presentation. Participants were eligible if they took part in at least one of 3 tasks: (1) aggregating or analyzing population-level data, (2) selecting cases for case reviews, or (3) abstracting data for individual case reviews. Of the 29 counties with OFR teams, we recruited participants from 2 counties—one corresponding to the dataset currently included in our study, and the other in anticipation of future data collection for the SMDC. Recruitment occurred from organizational listservs in response to informational emails sent by local OFR leadership, followed by an invitation to the voluntary and confidential survey. Participants provided consent and were enrolled via a link to the survey in a secure, web-based Research Electronic Data Capture (REDCap; Vanderbilt University) database [9,10].

We used the National Aeronautics and Space Administration-Task Load Index (NASA-TLX) survey to assess the cognitive workload of the contemporary OFR process for each task listed above that the OFR leader participated in [11]. The NASA-TLX is a validated, multidimensional tool that assesses a task’s subjective cognitive workload across 6 dimensions: mental demand, physical demand, temporal demand, performance, effort, and frustration [11]. The survey asks participants to rate each dimension on a scale between 1 and 20, with higher scores indicating greater perceived workload. For the performance dimension, lower scores correspond to higher perceived success [11]. Example items include “How mentally demanding was the task?” and “How hurried or rushed was the pace of the task?” Participants rated each applicable task they performed during the OFR process. Domain-specific mean scores were calculated rather than a composite overall score, as the NASA-TLX dimensions are designed to capture distinct aspects of workload rather than a single latent construct. Therefore, the mean and SD for each dimension were calculated.

Survey participants were then invited to a 30-minute semistructured interview with a research team member (MP). This interview approach combined a predefined structure with flexibility for follow-up questions based on participants’ responses [12]. The interview guide focused on participants’ responsibilities, workflows, mental workload, key data sources, and challenges. Survey findings were shared, and participants were asked to provide insight on the results. Wisconsin OFR training and technical assistance providers and a senior qualitative research expert (JDG) reviewed the guide to ensure its relevance and rigor. Interviews were conducted between October 2023 and January 2024 with participants providing verbal informed consent through secure, virtual conference software. The audio recording was transcribed and reviewed by the interviewer.

Interviews were stored and analyzed using MAXQDA 2024 (VERBI Software). The constant comparative method was used to analyze the transcripts, and inductive coding was applied to organize the information into emergent categories [13]. The codebook was repeatedly revisited and revised during the process [13]. When no new categories emerged from the analysis of additional interviews, code saturation was determined to be met. Using the final codebook, all interviews were coded by 1 researcher. Around 3 interviews were coded by a second researcher to assess intercoder reliability (ICR), exceeding the typical 10%‐25% double-coded interviews required to establish ICR [14]. The 2 researchers then compared coded segments, and disagreements were adjudicated.

### Phase 2: Data Dashboard Design and Evaluation

To mitigate data privacy concerns during dashboard development, a synthetic dataset was patterned on SDMC data [15]. The synthetic data were generated using the GPT-4 application programming interface (OpenAI) [16], with chain-of-thought instructions to create each variable from the data dictionary, similar to other best practices in prompt engineering [17]. The prompt incorporated aggregate cohort demographic descriptive statistics to preserve variable distributions within the SMDC dataset. Initial prompts were tested with 20 rows of patient data to evaluate the quality of the output before a dataset of 300 patients was created. This dataset was then scaled up to 1273 patients using YData Fabric (YData AI), a synthetic data generation software that employs generative adversarial networks to produce large volumes of data accurately replicating the statistical characteristics of the original data—in this case, the smaller synthetic dataset [18].

**Figure 1.**
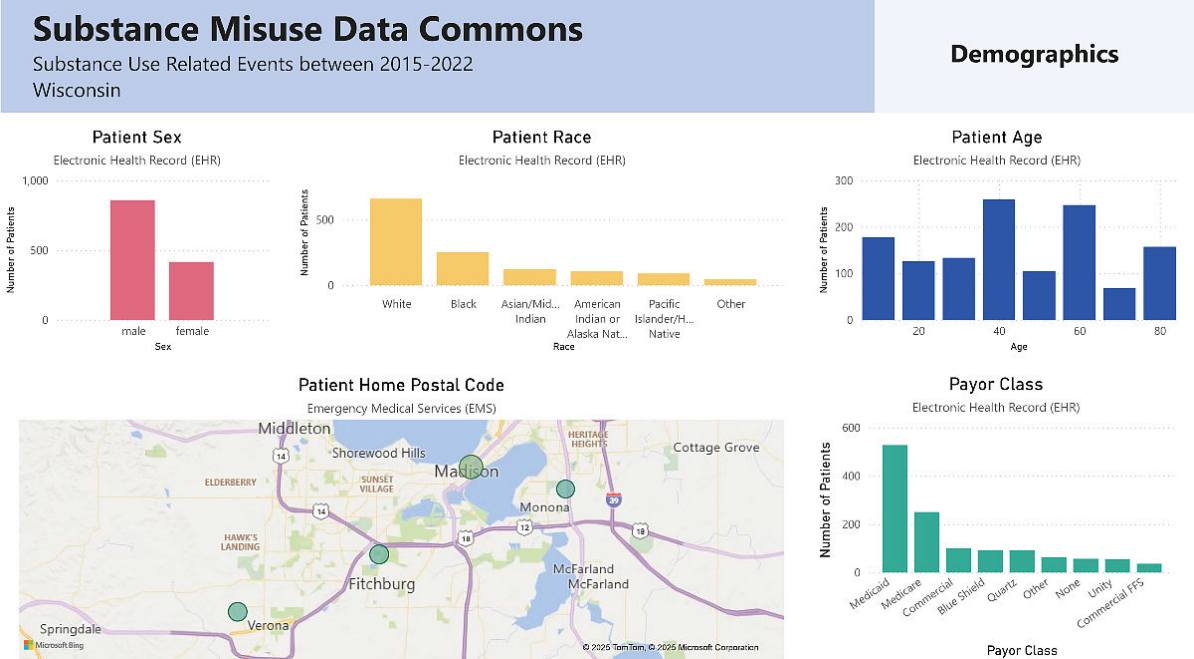
Substance misuse data dashboard: population-level visualizations.

**Figure 2.**
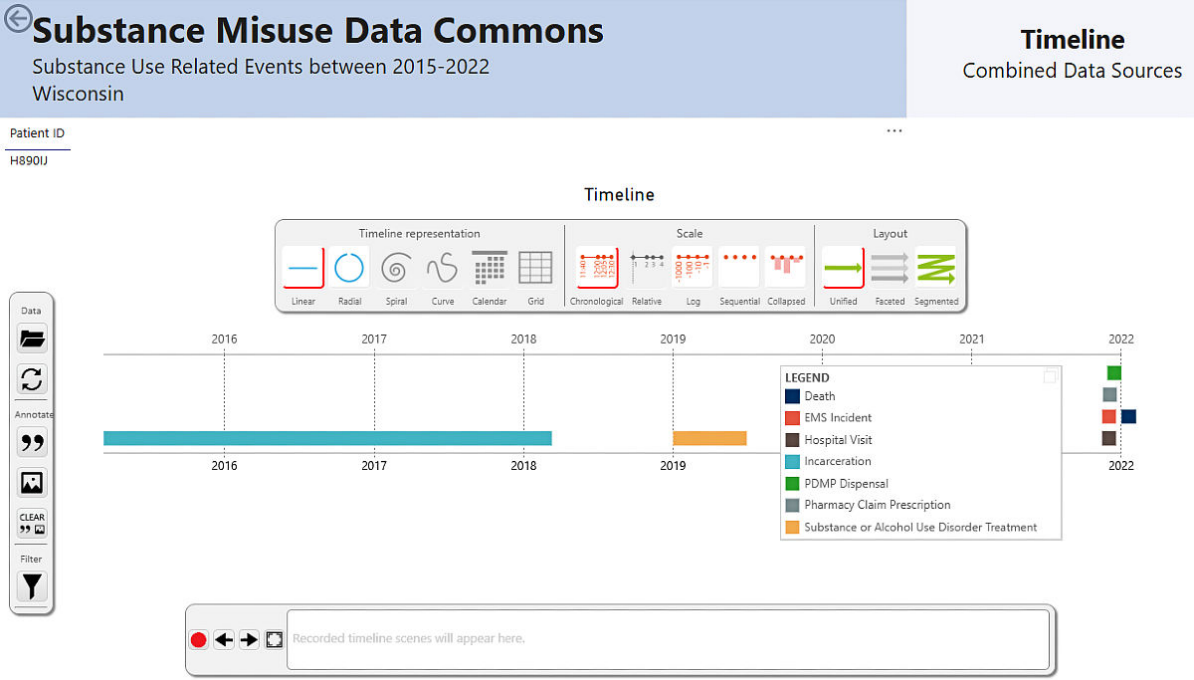
Substance misuse data dashboard: patient timeline visualizations.

An initial prototype of the dashboard was developed using Microsoft Power Business Intelligence (Microsoft 2024) [19]. The dashboard was designed to emulate the workflow and highlight the priority data sources identified in Phase 1. The dashboard consisted of 3 functional components: (1) visualizations of population-level data to identify demographics and trends (Figure 1); (2) line-level data, such as individual patient timelines, to facilitate case-based reviews (Figure 2); and (3) prediction tools, including census tract-level EMS patient incident predictions, deidentified hospital note topics, and a 30-day risk score for hospital readmission or death.

We conducted 2 iterative design sessions with emergency medicine physicians, including 2 EMS medical directors, a prehospital informatician, and a clinical human factors design expert. This team provided expertise in data visualization principles and linking hospital systems with prehospital emergency services, while allowing us to reserve OFR leaders for participation in the main study. The Heuristic Evaluation Checklist for Dashboard Visualizations was used to identify and address major usability issues [20]. These sessions refined the dashboard’s content, organization, and visual elements, culminating in a final prototype. After finalizing the prototype, a demonstration video was shared with participants to showcase its content, organization, and key features.

### Phase 2: Dashboard Design and Evaluation

End-user perceptions of the final prototype were assessed with semistructured interviews and surveys, using the same strategy and procedures to recruit OFR leaders as Phase 1. A total of 2 validated survey tools were used to evaluate end-user probability of adoption (Unified Theory of Acceptance and Use of Technology [UTAUT]) [21] and perception of usefulness (Perceived Usefulness Questionnaire) [22]. The interview guide explored participants’ perceptions of the dashboard, conditions for use, advantages, and suggested changes. One researcher coded all 7 interviews using the final codebook, and a second researcher independently coded 3 interviews to establish ICR. Disagreements were adjudicated. The mean and SD of all survey responses were calculated.

The full set of NASA-TLX, UTAUT, and Perceived Usefulness Questionnaire questions; our interview guides and codebooks; our synthetic dataset and prompts; and our demonstration video are all viewable in our GitLab repository [23].

### Ethical Considerations

The study followed the COREQ (Consolidated Criteria for Reporting Qualitative Research) reporting guidelines [24] (Checklist 1). This research was reviewed and approved by the Institutional Review Board at the University of Wisconsin, Madison (Institutional Review Board number 2023‐1091). Informed consent verbiage was included in our recruitment emails, and verbal consent was obtained during interviews. Participant email addresses were collected in the survey in order to invite the individual to participate in an interview. Survey and interview results were deidentified. No compensation was provided to participants.

## Results

### Phase 1: Analysis of Current Workflow

#### Surveys

**Figure 3.**
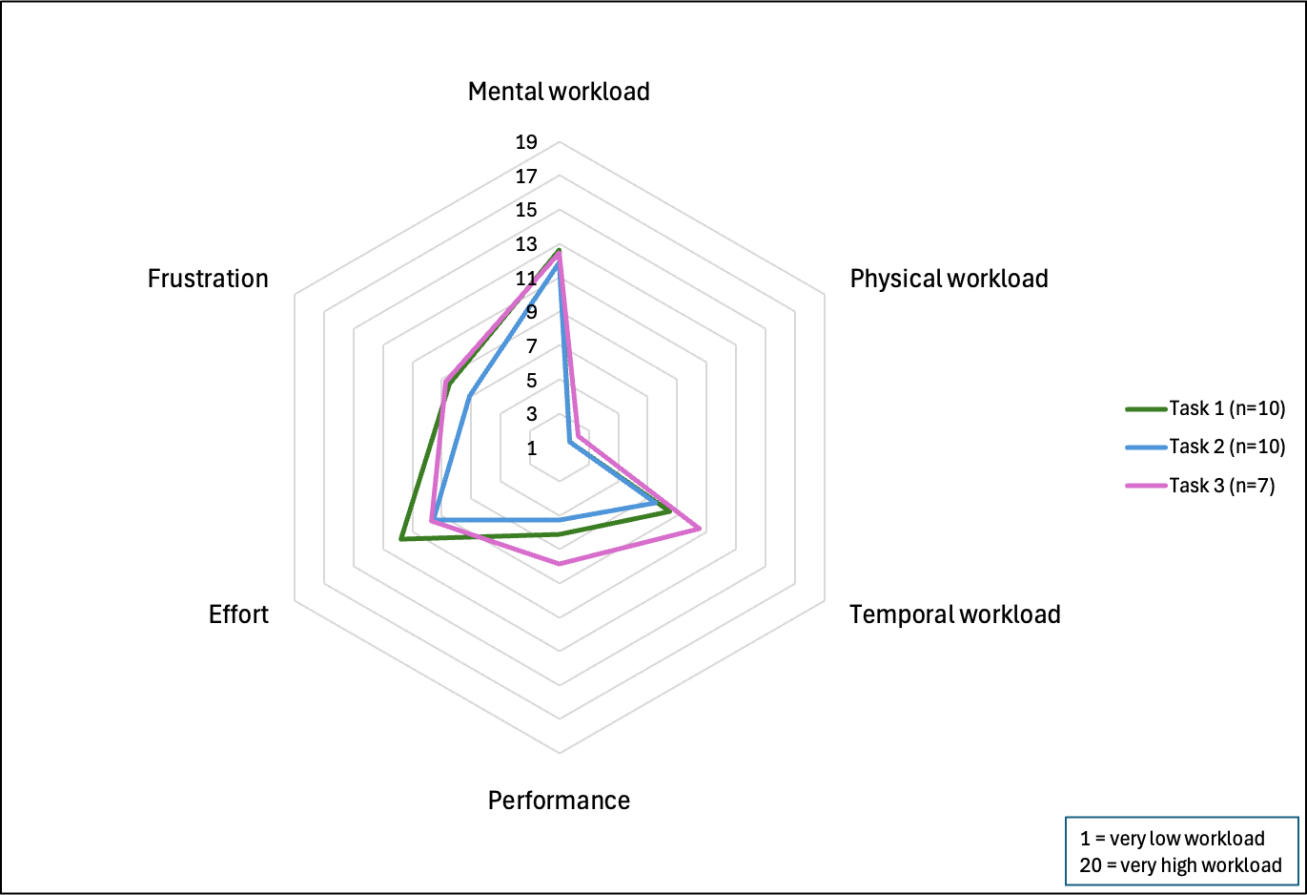
National Aeronautics and Space Administration-Task Load Index (NASA-TLX) survey results for overdose fatality review leaders in Wisconsin, 2023-2024.

A total of 11 OFR leaders, representing both county- and state-level agencies, participated in the survey. Table 1 provides the characteristics of the participants. The survey assessed the cognitive workload of 3 distinct tasks: (1) aggregating or analyzing population-level data, (2) selecting cases for case reviews, or (3) abstracting data for individual case reviews. Tasks had unequal sample sizes due to variations in task participation among participants. High mental workload was reported across all tasks (Figure 3). The time required for each task varied among participants. Per case review period of 1-3 months depending on the team, aggregating population-level data took an average of 5.5 hours (SD 3.09), selecting cases for case review averaged 7.95 hours (SD 5.67), and abstracting data for individual case reviews required an average of 10.50 hours (SD 8.86).

**Table 1.** Phases 1 and 2 survey demographics for overdose fatality review leaders in Wisconsin, 2023‐2024.

Demographic variables	Phase 1 participants (n=11)	Phase 2 participants (n=6)
Age (y), n (%)		
20‐29	5 (45.4)	2 (33.3)
30‐39	3 (27.3)	0 (0)
40‐49	2 (18.2)	3 (50)
50‐59	1 (9.1)	1 (16.7)
Sex, n (%)		
Female	10 (90.9)	6 (100)
Race, n (%)		
White	11 (100)	6 (100)
Education, n (%)		
Technical school, vocational training, community college	1 (9.1)	0 (0)
Bachelor’s degree	1 (9.1)	1 (16.7)
Master’s degree	9 (91.8)	5 (83.3)
Sector, n (%)		
Public health	9 (81.8)	6 (100)
Government	1 (9.1)	0 (0)
Education	1 (9.1)	0 (0)

#### Interviews

##### Key Data Sources

In total, 10 of the 11 participants completed qualitative follow-up interviews. Participants were asked to describe their data collection process, case selection methods, challenges, desires for a future state, and key data sources. Key OFR data sources highlighted by participants are presented in Table 2. Some of these sources were used for individual or population-level data only, but many were used for both. How these sources were used varied among participants. Summaries and selected quotes are highlighted below, while the full interview results and quotes are detailed in Multimedia Appendix 1.

**Table 2.** Key data sources for overdose fatality review leaders in Wisconsin, 2023‐2024.

Category	Data contributors and resources
High-impact data contributors	State vital records Next-of-kin interviews OFR^a^ agency partners
Key agency partners and contributors	ME’s^b^ or coroner’s office EMS^c^ DOC^d^ Law enforcement
Other data sources	Prescription Drug Monitoring Program Electronic Surveillance System for the Early Notification of Community-Based Epidemics First Watch (fire and rescue) Wisconsin Statewide Health Information Network Overdose Detection Mapping Application Program Consolidated Court Automation Programs Internet News Social media

a OFR: overdose fatality review.

b ME: medical examiner.

c EMS: emergency medical services.

d DOC: Department of Corrections.

##### Process

Participants described a typical workflow while preparing for an OFR meeting. Population-level data are analyzed to identify recent community trends. Next, representative cases are selected for review, and permission is obtained from local law enforcement to review the cases. OFR leaders then coordinate the collection of available and pertinent information on the decedents across multiple sources, which may include their own sources, agency partners, and next-of-kin interviews with the decedent’s family or friends. After compiling the data, they prepare a presentation for the case review meeting, often including a timeline of the decedent’s interactions with various agencies. During the sessions, OFR members, agency partners, and community representatives review the data and collaboratively brainstorm strategies for overdose prevention.

##### Case Selection

Participants reported several factors that influence case selection. About 50% (n=5) of the participants reported looking at demographic trends such as age, race, and sex. About 50% (n=5) of the participants reported combining multiple demographic and substance trends into a single theme and looked explicitly at decedents within that theme. For example, 1 participant stated, “Now that we’re doing theme selection, we may focus on a specific drug, like, I think the next theme that we’re doing is African American men between certain ages that historically used cocaine, but fentanyl was also involved in their cause of death.” Around 60% (n=6) of the participants prioritize selecting cases with comprehensive data, though they noted that this is challenging due to limited data availability during the initial selection process. Other key factors influencing case selection included obtaining permission from agency partners (n=5, 50%) and ensuring cases fell within jurisdictional boundaries (n=9, 90%).

##### Case Data Collection and Preparation

OFR leaders reported collecting data on decedents using publicly available databases and resources provided by their health departments and from the state-level data provided. They also reported requesting data from agency partners regarding any interactions with the decedent. After data are collected, they are processed manually by the OFR leader. One participant stated, “Then those individual partners have to go in, look at the specific case and then they have to like hand-put in all of the info and then they send those to me and then I scan them into our system so that we have them electronically, and then I have to take all of those electronic copies and upload those, one question at a time into REDCap.” After processing, all participants reported compiling the information into a timeline to display during case review presentations, which helped viewers to understand the decedent’s story.

##### Reported Challenges

When asked about challenges, 90% (n=9) of the participants identified a reliance on manual processes to collect data as a significant challenge. Due to limited bandwidths, responses to data requests from agency representatives are often delayed or incomplete, which impacts the preparation of case review materials. When final requests were not fulfilled, critical data were missing from presentations. Participants also reported several technological challenges, including siloed data sources (n=4, 40%), confusing data formatting (n=7, 70%), and other technological issues (n=10, 100%), all of which impacted data collection and preparation. As a result, 90% (n=9) of the participants highlighted time pressure as a major challenge for preparing for the OFR process. One participant explained this time pressure stating, “Lots of times for overdose fatality review, and this is true for me also, this is one part of my job, right? It’s part of my FTE, it is not my full FTE, so there are directions that I’m pulled for other projects that can sometimes limit the time that I have available to work on this.”

##### Desired Future State

Half (n=5) of the participants indicated that easier access to current data sources would be helpful, specifically mentioning simplified access as well as fewer lags to be able to identify and respond to current trends. One participant stated, “I think something that local public health and us specifically have always sort of struggled with is being able to keep up with that data…having it be a little easier for us to get local data and more quickly, that is an issue that is huge for us. We see things, and we hear about these trends, but we don’t necessarily always know that that’s happening until all of a sudden, it’s like, hey, we’re seeing this, you know, all across the county, and is that something we could have caught sooner had we been able to access that data quicker.”

Participants mentioned that additional data would help them. About 70% (n=7) of the participants desired health care data, most commonly substance use disorder treatment data; however, these data are protected by federal statute, which adds complexity to accessing and sharing them. About 50% (n=5) of the participants desired criminal justice data, whether they did not have access to it or did not often receive it when requested. Participants mentioned that they worked with multiple law enforcement agencies and regularly received data from some but not others. Additionally, 2 participants mentioned that they had not been able to perform next-of-kin interviews due to barriers in setting up interviews. Other desired data sources included childhood information from school districts or Child Protective Services and input from local organizations.

About 80% (n=8) of the participants mentioned that more collaboration or support would help the OFR process. Other participants specifically mentioned that increased collaboration between health departments and local agencies would help their current workflow, create the possibility to expand their services, and address long-term sustainability of OFR.

### Phase 2: Data Dashboard Design and Evaluation

#### Dashboard Design

The synthetic data dashboard prototype, developed based on Phase 1 data, was refined into a high-fidelity prototype for further usability and human factors evaluation. The final prototype featured 9 theme-based aggregate data pages covering demographics, substances used, health care interactions, prehospital emergency services, social and economic factors, mortality, prescription patterns, and treatment and recovery. Select components of the line-level data and timeline are shown in Figures1  2, and a full demonstration video is available in our GitLab repository [23]. The final dashboard prototype included advanced filtering capabilities, enabling users to refine data by specific time frames, substances, and death status. A total of 3 machine learning tools were integrated into the dashboard. First, the Hospital Note Topics Tool utilized latent Dirichlet allocation for topic modeling of EHR notes [25]. This tool identified prevalent themes and trends, such as patterns of substance use, health care utilization, and social determinants of health, providing users with a high-level understanding of key insights from unstructured text data. Second, the 30-Day Risk Score for Readmission and Death employed an eXtreme Gradient Boost machine learning model, which analyzed a combination of EHR notes and tabular data along with EMS and neighborhood census data to predict the likelihood of hospital readmission or death within 30 days. Third, the EMS Geographic Prediction Tool combined EMS response data with neighborhood-level census tract information to identify geographic areas at higher risk for overdose events. Additionally, the final prototype incorporated a drill-down feature, allowing users to filter patient populations by category and narrow them down to individual patients.

#### Surveys

**Figure 4.**
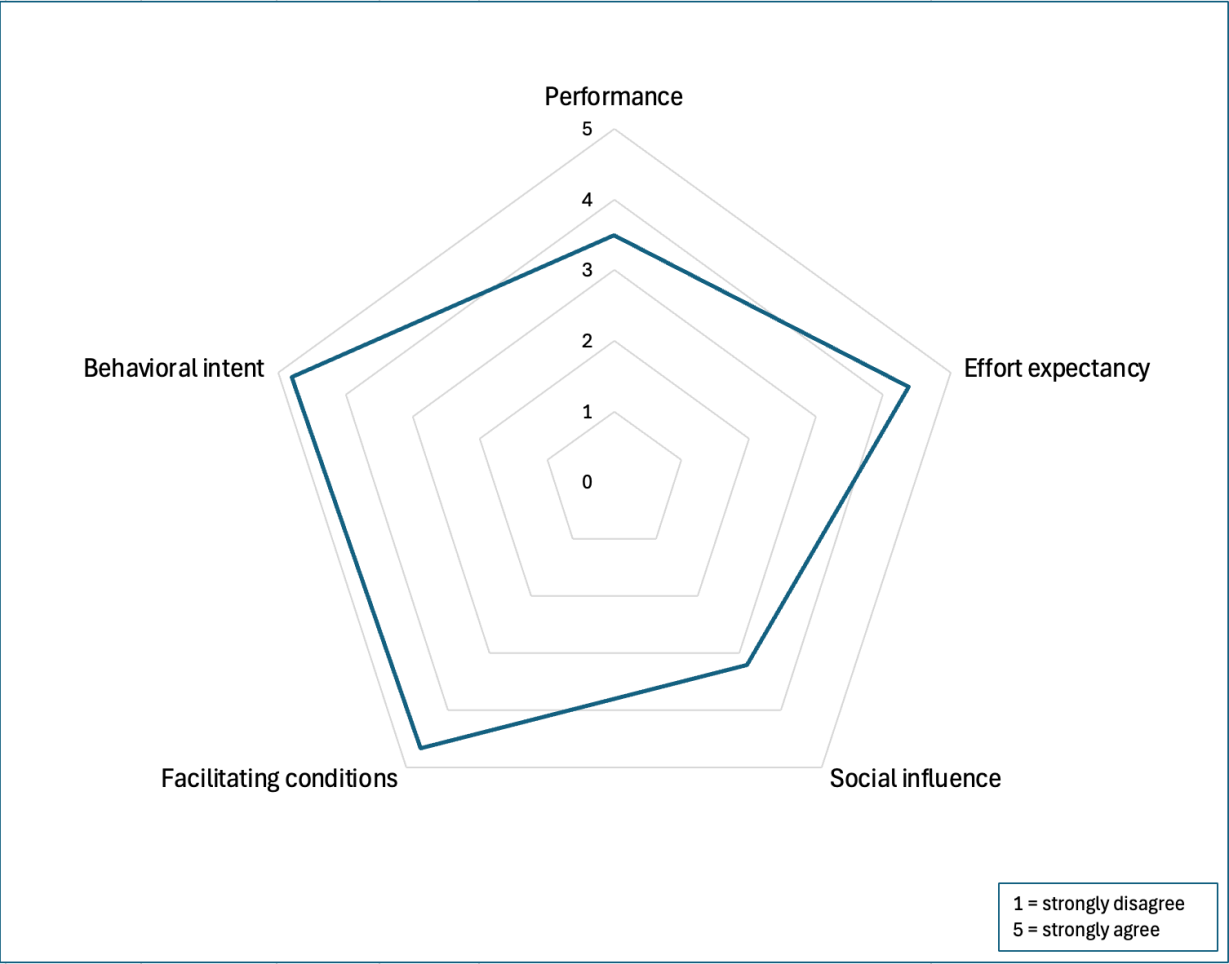
Unified Theory of Acceptance and Use of Technology (UTAUT) survey results for overdose fatality review leaders in Wisconsin, 2024.

**Figure 5.**
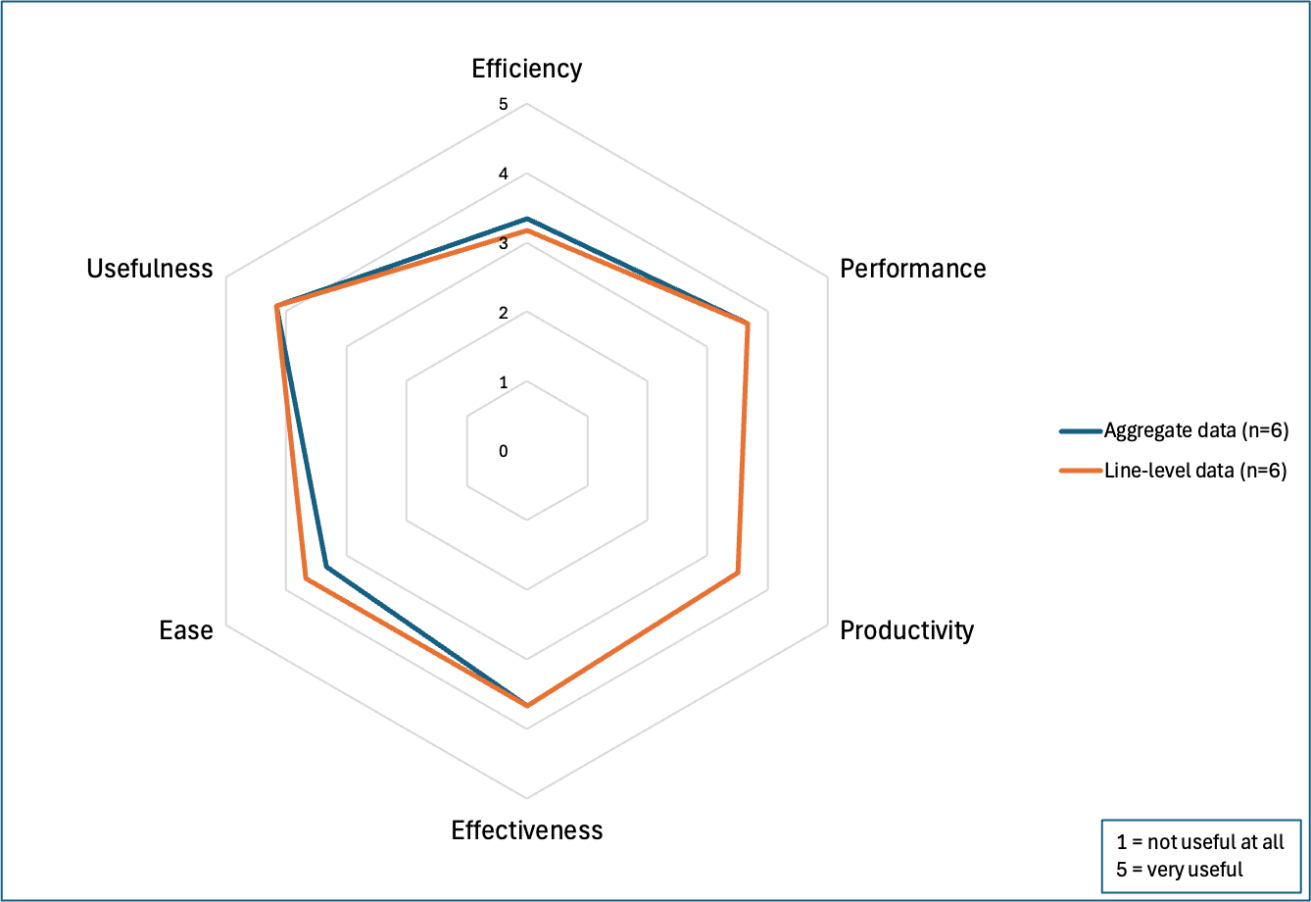
Perceived usefulness questionnaire results for overdose fatality review leaders in Wisconsin, 2024.

A total of 6 OFR organizers participated in the Phase 2 survey, representing stakeholders from county, state, and federal agencies. Additional demographic details are provided in Table 1. All the 6 participants reported analyzing aggregate data and selecting review cases, while 4 were involved in abstracting data for case reviews. The UTAUT results indicated that the dashboard would likely be adopted if made available to participants (Figure 4), with a mean of 4.07 out of 5.00 (SD 0.65). The Perceived Usefulness Questionnaire results suggested a moderately positive perception of usefulness for the aggregate and individual-level data (Figure 5), with means of 3.61 (SD 0.82) and 3.64 (SD 0.85) out of 5.00, respectively.

#### Interviews

A total of 7 OFR leaders participated in semistructured interviews. Participants were specifically asked about the potential benefits of the dashboard as well as areas for improvement. The summaries of the findings are outlined below, with detailed results and quotes provided in Multimedia Appendix 2.

##### Benefits of the Dashboard

All the participants (n=7) highlighted improved data access as a key benefit of the dashboard. This included expanding access to currently unavailable data sources, facilitating quicker and easier access to existing data, and increasing access for less-resourced communities. Additionally, 86% (n=6) of the participants indicated that the dashboard could help to optimize workflows by reducing the manual processes and introducing time-saving features. One participant stated, “That’s helpful in just kind of like streamlining it all in one spot, because the data that I do get like from emergency department visits, I have to go through every single entry, and then like, figure it out from there. So, this is quite nice to just have it in one spot where I can look at it, write it down, we’re done.” About 71% (n=5) of the participants noted that the dashboard’s tools are especially valuable for organizational outreach efforts. The machine learning tools, particularly the EMS geographic prediction tool and the 30-day risk score for readmission or death, were highlighted as most useful for these initiatives. All the participants (n=7) reported that the dashboard appeared easy to navigate, with an intuitive structure and organization. About 71% (n=5) of the participants specifically mentioned the ability to filter patients for specific populations as a standout feature, distinguishing the dashboard from other available tools.

##### Areas for Improvement

Most participants suggested adding more data sources. Specific recommendations included sexual orientation and gender identity, medical comorbidities such as chronic pain, and presenting data as rates instead of counts to better represent minority group trends. Concern about the accuracy and timeliness of the data was expressed by 71% (n=5) of the participants. The SMDC cohort, which is limited to patients with hospital encounters linked to other data, excludes individuals seen only by EMS or those without EMS or hospital contact. One participant expressed this concern, stating, “I like the way that it’s laid out, but because it’s only people who are going to the ED, I don’t know that we can draw conclusions about folks that are at risk of overdose, generally speaking.” The current data were also identified as a critical need for the dashboard. Participants proposed several technological enhancements to improve the dashboard’s usability. Specifically, 43% (n=3) of the participants suggested the ability to export data from the dashboard, while 57% (n=4) recommended adding other technological features such as additional filters and hover-over tips for ease of use. Other important but less commonly cited concerns included a training or learning curve (n=3, 43%), having jurisdictional access (n=2, 29%), adding to stigma or bias (n=2, 29%), having too many years aggregated in the dataset to be reflective of current trends (n=2, 29%), and the dashboard data being deidentified and therefore not being able to be connected to data about individuals from other sources (n=2, 29%).

## Discussion

### Principal Findings

This study is the first to systematically assess the cognitive workload of OFR leaders and apply those findings to the design of a user-centered data dashboard prototype. Using validated instruments (NASA-TLX, UTAUT, and Perceived Usefulness Questionnaire) and qualitative interviews, we identified substantial cognitive demands associated with data aggregation, case selection, and abstraction across multiple agencies. Guided by these findings, we developed and tested a prototype dashboard using synthetic data modeled on the SMDC to streamline data access and visualization. Participants reported improved usability, reduced time burden, and high potential for adoption in future OFR workflows.

### Interpretation and Implications

Our results highlight that OFR leaders face high mental and temporal demands due to fragmented data sources and reliance on manual processes, a challenge consistent with prior studies on multiagency data integration and decision-making in public health surveillance [26]. Previous dashboards have been implemented at the state or regional level to visualize aggregate overdose trends, yet few, if any, have incorporated both population- and individual-level data or been explicitly designed around the OFR workflow. By linking health system, emergency response, and correctional datasets within a privacy-preserving synthetic environment, our approach extends beyond traditional epidemiologic dashboards to directly support operational review teams. The integration of predictive modeling tools, such as 30-day readmission and EMS geographic risk scoring, further distinguishes this work from prior descriptive dashboards by introducing prospective, data-driven decision support [7,8]. These findings align with recent public health informatics initiatives emphasizing human-centered design, cognitive workload reduction, and automation to improve decision-making efficiency [27,28].

Our SMDC data dashboard serves as a comprehensive tool for extracting, transforming, and visualizing overdose data. It was designed to refresh with recent case information from multiple sources; incorporate automation for case matching, data formatting, and quality checks; and offer easy navigation to streamline current workflows. The dashboard helps automate the current workflow challenge of siloed data sources and includes additional data that participants found valuable. It integrates key data variables from multiple agencies, with options to filter the data by important demographic and substance-related factors. The automated timeline feature compiles data from all sources in our dataset, visually representing the events leading to an overdose death. Phase 2 participants saw the dashboard’s potential to reduce time pressure and reliance on manual processes. Additionally, in larger cities where reviewing every case is impractical or impossible, OFR leaders typically hand-pick cases to analyze in detail. By linking all cases and presenting the data in aggregate, this system enhances the understanding of overall trends and helps to better inform recommendations.

Many participants indicated that they would use this dashboard as an additional tool, rather than replacing their current methods. This likely explains our Phase 2 survey findings, which revealed a discrepancy between the tool’s effectiveness and efficiency. This preference highlights a key concern raised in Phase 2 interviews that cannot be resolved in future dashboard versions: the deidentified nature of the SMDC data prevents integration with other data sources, such as next-of-kin interviews, a crucial part of the case review process. Other areas of improvement discussed in Phase 2 interviews can be resolved.

Phase 1 participants emphasized a desire for increased collaboration and support. Some mentioned this in the form of improved relationships between the health department and other agencies. Expanding the sectors involved in OFR processes and broadening the information available for case reviews may highlight previously unseen gaps in care. Emergency department utilization is common among those who misuse opioids and other drugs, and the number of emergency department visits is associated with an increased risk of drug overdose [29]. Our dashboard aims to establish and improve data sharing between OFR teams and health care systems, which has been identified as an important prevention strategy implementation [30].

Our findings underscore the potential of informatics-driven tools to enhance collaboration among public health, health care, and community agencies. By reducing cognitive workload and manual data handling, such tools can accelerate case review preparation, standardize data access across jurisdictions, and allow OFR teams to focus more on interpretation and prevention strategies. The use of synthetic data generated through large language models also represents an innovative method for tool development when working with sensitive, multiagency datasets. As counties nationwide expand their OFR infrastructure, the approach demonstrated here offers a transferable model for building and evaluating data dashboards that are secure, scalable, and adaptable to local contexts.

### Limitations

The general process reported by participants in the interviews is mainly consistent with the process in the OFR Practitioner’s Guide and the Public Health and Safety Team toolkit, which are guiding frameworks for health departments when creating OFRs and holding case review meetings [31]. However, the feedback in this study was collected from local OFR leaders in Wisconsin, and therefore, these findings may not be generalizable to other systems. Limitations inherent to the SMDC include its inclusion criteria and its deidentified nature. All data variables included in the prototype dashboard are included in the SMDC; however, the SMDC does not include all data variables important to OFR leaders when presenting cases. Finally, addressing challenges in the OFR process with new technology may not ultimately lead to better outcomes. Substance misuse is both a complex medical condition and an evolving public health issue. In order to make meaningful progress and improve outcomes, sustained collaboration across health care systems, public health agencies, and communities will be essential to reform policies, reduce disparities, and improve medical care.

The existing OFR process is built on a thorough, team-based approach, but it includes several cognitively demanding tasks, and there are multiple challenges to timely data preparation. Increased collaboration, access to standard, centralized tools, and comprehensive data could build upon the rigorous work already being done by OFR teams in order to further augment and automate workflows to reduce manual work. We designed a user-centered data dashboard to help reduce the cognitive workloads identified from surveys and incorporate the desired data sources and workflows gathered from the interviews. Evaluative feedback indicated many potential benefits as well as some areas for improvement. This insight will guide the development of a real-time data dashboard accessible to OFR leaders in their review process.

### Conclusions and Broader Implications

Despite these limitations, this study demonstrates a replicable, human-centered approach for modernizing OFR workflows through data integration and visualization. The combination of cognitive workload analysis, synthetic data modeling, and dashboard-based decision support provides a foundation for scalable OFR modernization across states. Beyond overdose prevention, this framework illustrates how human factors and data science methods can be combined to enhance other multidisciplinary public health review processes. Future work will focus on deploying the dashboard with live SMDC data, expanding data sources, and assessing real-world impacts on timeliness, data completeness, and prevention outcomes.

## Supplementary material

10.2196/79407 Multimedia Appendix 1 Phase 1 interview results for overdose fatality review leaders in Wisconsin, 2023-2024.

10.2196/79407 Multimedia Appendix 2 Phase 2 interview results for overdose fatality review leaders in Wisconsin, 2024.

10.2196/79407 Checklist 1 COREQ checklist.

## ABBREVIATIONS

COREQ: Consolidated Criteria for Reporting Qualitative Research
EHR: electronic health record
EMS: emergency medical services
ICR: intercoder reliability
NASA-TLX: National Aeronautics and Space Administration-Task Load Index
OFR: overdose fatality review
REDCap: Research Electronic Data Capture
SMDC: Substance Misuse Data Commons
UTAUT: Unified Theory of Acceptance and Use of Technology
